# Astragalus Root and Elderberry Fruit Extracts Enhance the IFN-β Stimulatory Effects of *Lactobacillus acidophilus* in Murine-Derived Dendritic Cells

**DOI:** 10.1371/journal.pone.0047878

**Published:** 2012-10-30

**Authors:** Hanne Frøkiær, Louise Henningsen, Stine Broeng Metzdorff, Gudrun Weiss, Marc Roller, John Flanagan, Emilie Fromentin, Alvin Ibarra

**Affiliations:** 1 Department of Veterinary Disease Biology, Copenhagen University, Frederiksberg, Denmark; 2 Naturex SA, Avignon, France; 3 Naturex Spain, Quart de Poblet, Spain; 4 Naturex Incorporated, South Hackensack, New Jersey, United States of America; Friedrich-Alexander-University Erlangen, Germany

## Abstract

Many foods and food components boost the immune system, but little data are available regarding the mechanisms by which they do. Bacterial strains have disparate effects in stimulating the immune system. Indendritic cells, the gram-negative bacteria *Escherichia coli* upregulates proinflammatory cytokines, whereas gram-positive *Lactobacillus acidophilus* induces a robust interferon (IFN)-β response. The immune-modulating effects of astragalus root and elderberry fruit extracts were examined in bone marrow-derived murine dendritic cells that were stimulated with *L. acidophilus* or *E. coli*. IFN-β and other cytokines were measured by ELISA and RT-PCR. Endocytosis of fluorescence-labeled dextran and *L. acidophilus* in the presence of elderberry fruit or astragalus root extract was evaluated in dendritic cells. Our results show that both extracts enhanced *L. acidophilus*-induced IFN-β production and slightly decreased the proinflammatory response to *E. coli*. The enhanced IFN-β production was associated with upregulation of toll-like receptor 3 and to a varying degree, the cytokines IL-12, IL-6, IL-1β and TNF-α. Both extracts increased endocytosis in immature dendritic cells, and only slightly influenced the viability of the cells. In conclusion, astragalus root and elderberry fruit extracts increase the IFN-β inducing activity of *L. acidophilus* in dendritic cells, suggesting that they may exert antiviral and immune-enhancing activity.

## Introduction

Foods and food components with immune-boosting activity are constantly sought after [1]. Some of the most promising components are probiotic bacteria, which stimulate the immune system on oral administration [2], including *Lactobacillus* strains. Certain lactobacilli induce interferon (IFN)-β and interleukin (IL)-12 production in dendritic cells, which leads to upregulation of type 1 helper T cells (Th1) [3].IFN-β induces IL-12, TLR3 and other genes that mediate viral defense—culminating in robust stimulation of the adaptive immune system [4]. In addition to its strong antiviral effects and regulation of innate and adaptive immunity [4], IFN-β has anti-inflammatory properties, upregulating IL-1β receptor antagonist [5], which mitigates the proinflammatory effects of IL-1β [6]. Accordingly, the stimulation of the IFN-βproduction in DCs may boost the immune systems while simultaneously leading to reduction in low-grade inflammation in the tissue.

In contrast *to L.acidophilus*, although the gram-negative *Escherichia coli* strain Nissle 1917 also leads to IL-12 production, albeit at lower levels, it does not induce significant IFN-β production or upregulation of viral defense genes [3]. Thus, the induction of IL-12 may take place by two distinct pathways, indicating the importance of the system used to assess the immunomodulatory activity of bioactive food or food compounds. Dendritic cells are professional antigen-presenting cells that respond to a microbial signal through pathogen recognition receptors, such as toll-like receptors (TLRs), and orchestrate the responses of other immune cells [7], [8]. Thus, dendritic cells are model cells that can be used to assess the immune-modulating activities of food and food components. The responses that are generated in dendritic cells, particularly with regard to surface marker expression and cytokine production, depend on the cellular events that are induced by specific microorganisms [9].

Many of the claims for the immune-modulating activities of plants and plant components originated in traditional medicine, and only recently have such mechanisms been researched [10]. Reducing chronic inflammation in tissues and enhancing immune responses during viral and bacterial infections using food and food components, including complementary and alternative medicine, has received growing interest [11]. Astragalus, *Astragalus propinquus* S. syn. *Astragalus membranaceus* F. (Family Fabaceae), is a flowering plant that exists worldwide. Its membranous milk-vetch roots are used in traditional Chinese medicine as a powder or in a decoction to ameliorate general weakness and increase overall vitality [12]. Astragalus is being investigated with regard to its immune-stimulating properties. In human peripheral blood mononuclear (PBM) cells, astragalus root extract induces monocyte maturation [13].

Chemically, astragalus comprises polysaccharides, saponins, flavonoids (including calycosin and formononetin), amino acids, and trace elements. Saponins are primarily astragalosides [14]. Polysaccharide fractions of Astragalus root extract have also been isolated [14]–[17]. Whereas certain plant polysaccharides induce maturation of dendritic cells [18], they have not been reported to upregulate the Th1-polarizing cytokines IFN-β and IL-12. In general, low-molecular-weight compounds from plants are incapable of stimulating antigen-presenting cells, but they are often potent modulators of microbially induced antigen presentation [19], [20].

Elderberry, *Sambucus nigra* (Family Adoxaceae), is a shrub that grows in moist soil, bearing stems that reach up to 4 m high, and is found in Europe, west and central Asia, and north Africa. Its fruits are black-purple, edible, berry-like drupes [21] that have been traditionally used for their antiviral, antibacterial, and anti-inflammatory properties [21], [22], the primary constituents of which are flavonoids. Other major secondary metabolites include triterpenes, sterols, phenolic acids and their corresponding glycosides, and essential oil [22]. Several lectins have also been isolated from elderberry [23]–[25].

Although immune-modulating and -stimulating properties of specific probiotic bacteria and herbal extracts have been demonstrated, the effects of herbal extracts hitherto have been investigated by stimulation with LPS or other microbial ligands or plant derived mitogens and, to our knowledge no studies have reported the effects of probiotics and plant extracts in combination. As a result of a screening of the immunomodulatory activity of a large number of plant extracts we identified two extracts from astragalus root and elderberry, respectively, that were especially potent in enhancing the IL-12 production induced by *L.acidophilus*, while they reduced the IL-12 production induced by *E.coli*. Accordingly, in the present study we aimed to investigate the immunomodulatory effect of astragalus root and elderberry fruit extracts in dendritic cells that were stimulated with *L. acidophilus* or *E. coli*. We hypothesized that the disparate effects of the extracts on the two bacteria were due to an enhancing effect on the IFN-β response, which is almost absent in *E.coli* stimulated DCs [3].

## Materials and Methods

### Astragalus root and elderberry fruit extract preparation

Astragalus root and elderberry fruit extracts were produced in an industrial process at Naturex Inc, USA (Astragalus root extract reference: ED161024, Lot number: D184/012/D10 with a content of 50% Gum arabicum. Elderberry fruit extract reference: EA145100, batch number: 231/55/A9 with 65% maltodextrin as carrier material). Both extracts were analyzed at AINIA (Valencia, Spain) per the norms in vigor for microorganism content, including aerobic mesophiles (UNE-EN ISO 4833:2003), yeast, fungi (European pharmacopoeia 6.7, part 2.6.12), enterobacteriaceae (ISO 21528-2:2004), *E. coli* (ISO 21528-1:2004), *Staphylococcus* spp. (UNE EN-ISO 6888-1:2000), and *Salmonella* spp. (UNE EN-ISO 6579:2003); pesticides (European pharmacopoeia); and heavy metals (Cd, Pb, Hg, Ar). The microorganism content in both extracts did not exceed 1×10^3^ CFU per g, and the amounts of pesticides and heavy metals were below the maximum residue limits [26].

For all analyses, 50 mg of the lyophilized samples was added to 5 mL deionized sterile water and incubated for at least 30 min on a shaker and centrifuged at 400 *g* for 10 min. The supernatants were transferred to new tubes and assayed on the same day.

### Bacterial strains, growth conditions, and preparation of UV-killed bacteria

The gram-positive lactic acid bacterium *Lactobacillus acidophilus* NCFM (Danisco, Copenhagen, Denmark) was grown anaerobically overnight at 37°C in de Man Rogosa Sharpe (MRS) broth (Merck, Darmstadt, Germany) and subcultured twice. Cells were harvested by centrifugation at 2000 *g* for 15 min, washed twice in phosphate-buffered saline (PBS, Bio Whittaker, East Rutherford, NJ, USA), and resuspended in 1/10 of the growth volume of PBS. Because they affect the same stimulatory patterns as live bacteria, UV-killed bacteria were used to avoid growth during dendritic cell stimulation and ensure reproducibility. The bacteria were killed in a 20-min exposure to UV light.

*Escherichia coli* Nissle 1917 O6:K5:H1 (Statens Serum Institut, Copenhagen, Denmark), a gram-negative probiotic bacterium, was grown aerobically overnight at 37°C in Luria-Bertani (LB) broth (Merck) and killed by a 45-min exposure to UV light. The bacteria were stored at −80°C, their concentration was calculated as the content of dry matter per mL on lyophilization, and the dry weight was corrected for buffer salt content. The absence of viable cells was verified by plating the UV-exposed bacteria on MRS and LB agar.

### Generation of murine dendritic cells

Bone marrow-derived dendritic cells were prepared as described [9]. Briefly, bone marrow from C57BL/6 mice was flushed from the femur and tibia and washed twice in sterile PBS. Bone marrow cells (3×10^5^) were used to seed Petri dishes in 10 mL RPMI 1640 (Sigma-Aldrich, St. Louis, MO), containing 10% (v/v) heat-inactivated fetal calve serum that was supplemented with penicillin (100 U/mL), streptomycin (100 µg/mL), glutamine (4 mM), 50 µM 2-mercaptoethanol (all purchased from Cambrex Bio Whittaker, Charles City, IA), and 15 ng/mL murine granulocyte-macrophage colony-stimulating factor (GM-CSF) added as <1% culture supernatant harvested from the GM-CSF-tranfected Ag8.653 cell line [27]; which was kindly provided by Dr. R. Tisch (University North Carolina, Chapel Hill, NC).

All animals used as source of bone marrow cells were housed under conditions approved by the Danish Animal Experiments Inspectorate (Forsøgdyrstilsynet) and experiments were carried out in accordance with the guidelines of ‘The Council of Europe Convention for the Protection of Vertebrate Animals used for Experimental and other Scientific Purposes’. Since the animals were employed as a source of cells, and that no animals were used for the experiments, no specific approval was required for this study. Hence, the animals used for this study are included in the general facility approval for the faculty of Life Science, University of Copenhagen.

The cells were incubated for 8 days at 37°C in a 5% CO_2_ humidified atmosphere. On Day 3, 10 mL of complete medium, containing 15 ng/mL GM-CSF, was added. On Day 6, 10 mL of medium was removed and replaced with fresh medium. Nonadherent immature dendritic cells were harvested on Day 8 and examined for purity by CD11c immunostaining and flow cytometry.

### Immunostaining and flow cytometry

Dendritic cells were harvested and resuspended in cold PBS that contain 1% (v/v) fetal bovine serum, 0.15% (w/v) sodium azide (PBS-Az), and anti-mouse FcγRII/III (3 µg/mL, BD Biosciences, San Jose, CA, USA) to block the nonspecific binding of antibody reagents. The cells were stained with phycoerythrin (PE)-conjugated anti-mouse MHCII, allophycocyanin (APC)-conjugated anti-mouse CD86, and PE-conjugated anti-mouse CD11c (Southern Biotech, Birmingham, AL) and analyzed on a BD FACS array flow cytometer (BD Biosciences) in 10,000-cell counts. The geometric mean fluorescence intensity (MFI) was determined, reflecting the level of expression. To assess the viability of the cells on stimulation, 7-AAD (Pharmingen, CA, USA) was added to each sample for the last 10 minutes of the incubation with antibodies.

### Cell treatments and stimulation with bacteria

Immature dendritic cells (2×10^6^ cells/mL) were resuspended in fresh medium that was supplemented with 10 ng/mL GM-CSF, and 500 µL/well was used to seed 48-well tissue culture plates (Nunc, Roskilde, Denmark). Cells (10^6^) were treated with herbal extracts at final dilutions of the reconstituted extracts of 1∶18, 1∶60, 1∶180, and 1∶600 (corresponding to the concentrations 500, 150, 50 and 15 µg/mL, respectively).

Dendritic cells were treated with the herbal extract and incubated for 30 minutes prior to the addition of *L. acidophilus* or *E. coli*, which were suspended in medium and added (100 µL/well) to a final concentration of 10 µg/mL. Optimal bacterial concentrations were determined in a previous study [28]. The cell cultures were incubated at 37°C in 5% CO_2_ for 20 hr, and supernatants were harvested for cytokine quantification. In the PCR analysis experiments, the cells were harvested 2–10 hours after stimulation. The two carrier materials, gum arabicum and maltodextrin were tested for immunomodulatory effects in the cellular assay *per se*, but showed no immunomodulatory activity.

### RNA extraction

Murine dendritic cells were harvested after stimulation and homogenized using a QIAshredder (Qiagen, Ballerup, Denmark), and RNA was extracted using the MagMAX-96 Total RNA Isolation kit (AM1830, Applied Biosystem) on a MagMAX Express Magnetic Particle Processor (Applied Biosystem). RNA concentration was measured on a Nanodrop (Thermo, Wilmington, USA).

### Quantitative Real-Time PCR

One microgram of total RNA was reverse-transcribed using the TaqMan Reverse Transcription Reagent kit (Applied Biosystems, Foster City, USA) with random hexamer primers per the manufacturer's instructions. The resulting cDNA was stored in aliquots at −80°C. To design the primer and probe sequences, we retrieved the regions that encoded for the genes of interest from the GenBank EMBL databases: TLR-3 (NM_126166), IFN-β (NM_010510), IL-12 p40 (NM_008352), IL-10 (NM_010548), and beta actin (NM_007393). Primers and probes were designed using Primer Express 3.0 (Applied Biosystems) and tested for specificity using BLAST. HPLC-purified forward and reverse primers were manufactured by DNA Technology (Aarhus, Denmark).

The probes were labeled with the 5′ reporter 6-carboxy-fluorescein (FAM) and the 3′ quencher NFQ-MGB (Applied Biosystems). The sequences of the primers and probes are listed in Table 1. Primer and probe concentrations were optimized, and standard curves were generated for each set of primers and probe to determine the efficiency of the amplification for each dilution, (data not shown).

**Table 1:** Real-Time PCR primers and probes.

Target	Primers and probes	Sequences (5′-3′)
IFN-β (NM_010510)	Forward	CCCTATGGAGATGACGGAGAAG
	Reverse	GAGCATCTCTTGGATGGCAAA
	Probe	TGCAGAAGAGTTACACTGC
IL-12 p40 (NM_008352)	Forward	GCCAGTACACCTGCCACAAA
	Reverse	TGTGGAGCAGCAGATGTGAGT
	Probe	AGGCGAGACTCTG
IL-10 (NM_010548)	Forward	AGCATGGCCCAGAAATCAAG
	Reverse	CGCATCCTGAGGGTCTTCAG
	Probe	AGCATTTGAATTCCCTGGGT
β-actin (NM_007393)	Forward	CGATGCCCTGAGGCTCTTT
	Reverse	TGGATGCCACAGGATTCCA
	Probe	CCAGCCTTCCTTCTT

The amplifications were performed in 20 µL, containing 1×TaqMan Universal PCR Master Mix (Applied Biosystems), forward and reverse primer (concentration 900 nM each), 200 nM TaqMan MGB probe, and purified target cDNA. The cycling program comprised 20 sec at 95°C and 40 cycles of 3 sec at 95°C and 30 sec at 60°C, run on an ABI Prism 7500 (Applied Biosystems). Amplifications were performed in triplicate, and DNA contamination controls were included. The reactions were normalized to beta actin. Relative transcript levels were calculated per Pfaffl [29].

### Cytokine ELISA

After 20 hr of stimulation, culture supernatants were collected and stored at −80°C for cytokine analysis. Murine IL-12(p70), IL-10, IL-6, TNF-α, IL-1β, and IFN-β were analyzed using commercial ELISA kits (R&D Systems, Minneapolis, USA).

### Endocytotic activity

Fluorescein isothiocyanate (FITC)-conjugated dextran (10 kD) was obtained from Sigma-Aldrich (St. Louis, MO). *L. acidophilus* was labeled with Alexa Fluor 647 per the supplier's protocol. Briefly, 1 volume ofAlexa Fluor 647, solubilized in DMSO (5 mg/mL), was added to 9 volumes of bacteria in 0.1 M sodium carbonate buffer, pH 9. After 1 hour of incubation, the bacteria was washed 3 times, Dendritic cells were incubated with elderberry fruit or astragalus root extract or stimulated with 1 µg/mL lipopolysaccharide (LPS, *E. coli*, Sigma-Aldrich) prior to the addition of fluorescence-labeled dextran or *L. acidophilus*. Endocytosis was measured by flow cytometry and was expressed as the percentage of cells that endocytosed fluorescence-labeled dextran or *L. acidophilus* or the amount of endocytosed dextran/bacteria per cell, based on mean florescence intensity (MFI).

### Statistical analysis

Statistical analyses were performed by Prof. Alberto José Ferrer-Riquelme and Ass. Prof. José Manuel Prats-Montalbán, Multivariate Statistical Engineering Research Group, Department of Applied Statistics, Operations Research and Quality, Universidad Politécnica de Valencia, using SGWIN (Statgraphics Plus for Windows version 5; Statistical Graphics, Rockville, MD). The results of each experiment were analyzed by ANOVA. We assumed a type I risk α of 0.05, and the difference between groups was evaluated using the least significance difference (LSD) intervals (95% confidence level). The difference between 2 groups was considered significant when the LSD intervals did not overlap (p-value<0.05).

## Results

**Figure 1:**
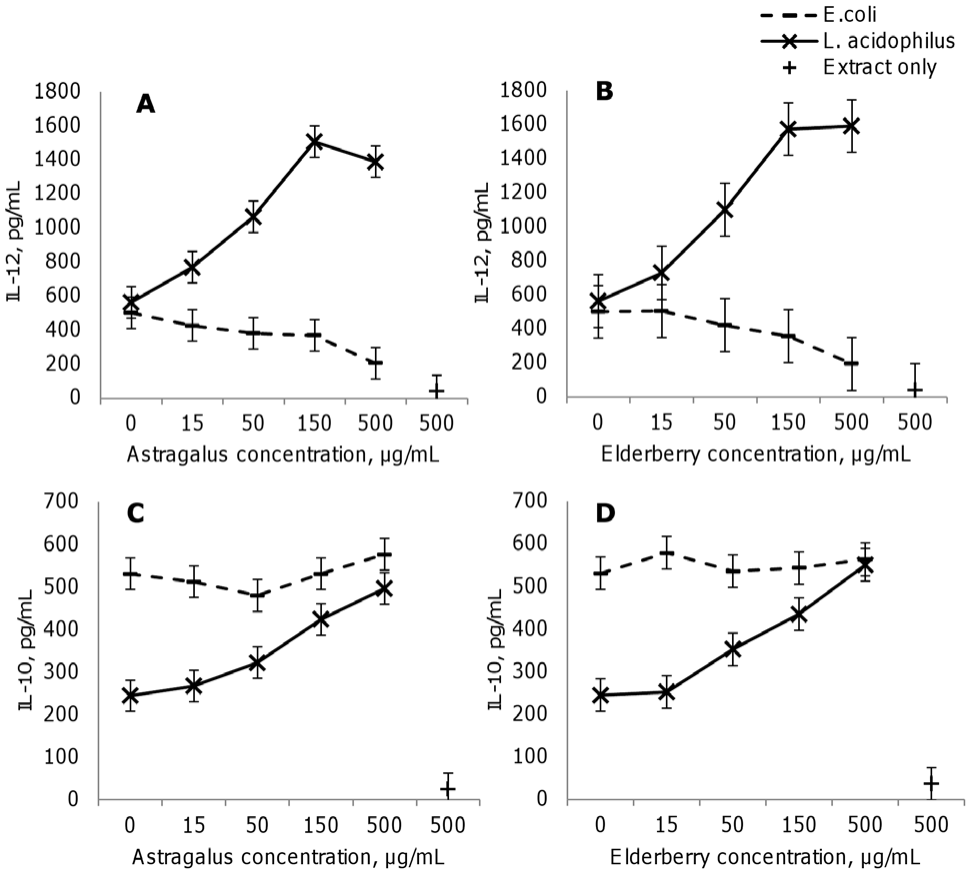
Astragalus and elderberry extracts modulate IL-12 and IL10 production in dendritic cells. Astragalus root (A, C) and elderberry fruit (B, D) extracts enhance IL-12 (A, B) and IL-10 (C, D) production when added to murine bone marrow-derived dendritic cells at concentrations from 15 to 500 µg/mL 30 min prior to stimulation with *L. acidophilus* (10 µg/mL) but not with *E. coli* (10 µg/mL). Supernatants were harvested for ELISA after 20 hr incubation. The results are representative of at least 3 experiments. Error bars are representative of the LSD intervals. The difference between 2 groups was considered significant when the LSD intervals did not overlap (p-value<0.05).

**Figure 2:**
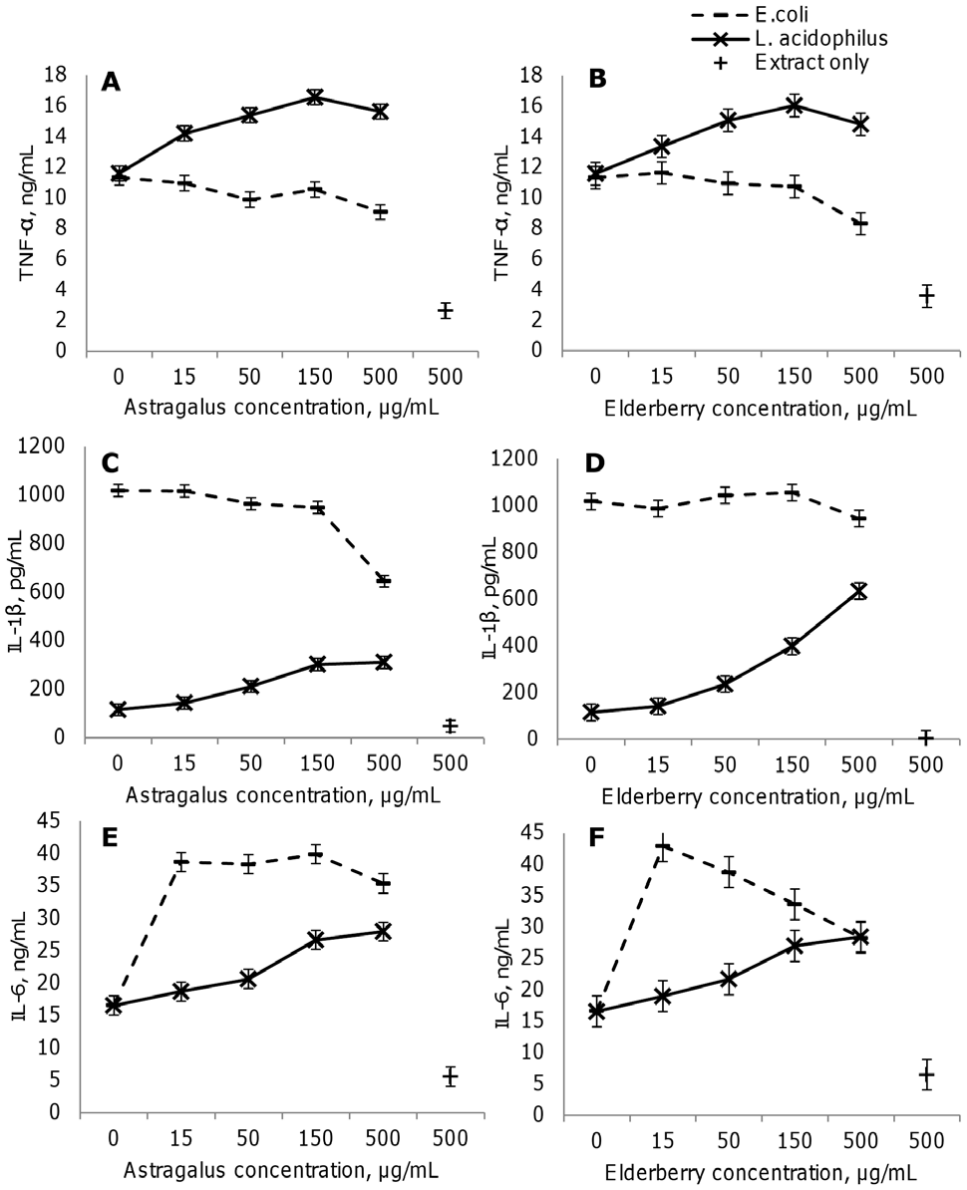
Astragalus and elderberry extracts modulate TNF-α, IL-1β, and IL-6 in dendritic cells. Astragalus root (A, C, E) and elderberry fruit (B, D, F) extracts differentially modulate TNF-α (A, B), IL-1β (C, D), and IL-6 (E, F) production when added to murine bone marrow-derived dendritic cells at concentrations from 15 to 500 µg/mL 30 min prior to the addition *L. acidophilus* (10 µg/mL) or *E. coli* (10 µg/mL). Supernatants were harvested for ELISA after 20 hr incubation. The results are representative of at least 3 experiments. Error bars are representative of the LSD intervals. The difference between 2 groups was considered significant when the LSD intervals did not overlap (p-value<0.05).

Extracts of astragalus root or elderberry fruit were added at dilutions 1∶18 to 1∶600 (corresponding to approximately 10, 50, 100 and 500 µg/mL dry matter, or from around 4 to 250 µg/mL pure extract) to dendritic cells 30 min before stimulation with *L. acidophilus* or *E. coli,s* or at 500 µg/mL to unstimulated cells. IL-12 (Figure 1A, 1B), TNF-α (Figure 2A, 2B) and IL-6 (Figure 2 E, 2F) levels increased in dendritic cells to the same extent on stimulation with *E. coli* and *L. acidophilus*. Conversely, IL-10 (Figure 1C, 1D) and IL-1β (Figure 2C, 2D) rose significantly higher with *E. coli* versus *L. acidophilus* (p<0.05). In immature dendritic cells, 500 µg/mL of extract did not affect IL-10 and IL-12 production and slightly increased IL-6 and TNF-α levels, albeit to a lesser extent than microbially induced cytokine production, except for IL-1β, which rose with elderberry fruit extract to comparable levels as with *L. acidophilus*, while astragalus root extract alone had no significant effects (data not shown).

After incubation with astragalus root or elderberry fruit extract and stimulation with *E. coli*, the production of IL-12 (Figure 1A, 1B) in dendritic cells fell dose-dependently, by 50% at the concentration 500 µg/mL. IL-10 production was unaffected by these extracts in *E. coli*-stimulated dendritic cells (Figure 1C, 1D).

In contrast, when the extracts were added prior to stimulation with *L. acidophilus*, IL-12 rose significantly. At the highest concentrations of extracts (50 to 500 µg/mL), IL-12 production increased by more than 2-fold significantly higher than the effects of *L. acidophilus* alone (p<0.05). Similarly, IL-10 levels climbed significantly—by 2-fold when *L. acidophilus* was incubated with astragalus root or elderberry fruit extract at 150 to 500 µg/mL (Figure 1C, 1D)) (p<0.05). The IL-12 stimulating effect of the two extracts lasted for at least up to two hours, while after 3 hours of incubation with extract prior to stimulation with *L.acidophilus*, no significant effect of the extract was observed (data not shown). The immune modulating effects were not due to physical interactions or intermolecular complex formation as washing the cells after incubation with plant extract and prior to stimulation with bacteria did not alter the immune modulatory profile of the extracts (data not shown).

The production of the proinflammatory cytokines TNF-α, IL-1β, and IL-6 is shown in Figure 2. Whereas the extracts inhibited *E. coli*- and increased *L. acidophilus*-induced TNF-α slightly (p<0.05, Figure 2A, 2B), they had robust effects on IL-1β and IL-6. Notably, *L. acidophilus*-induced IL-1β levels were enhanced significantly—up to 6-fold when dendritic cells were coincubated with 500 µg/mL elderberry fruit extract (p<0.05, Figure 2D) and up to 3-fold when coincubated with 500 µg/mL astragalus root extract (p<0.05, Figure 2C).

**Figure 3:**
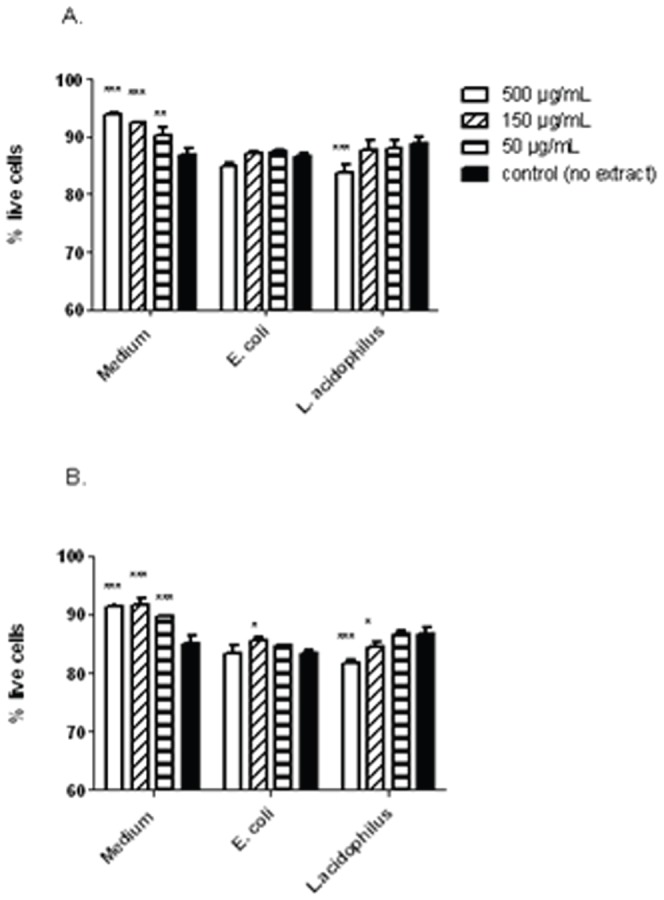
Astragalus root and Elderberry extracts only slightly affect the viability of DCs. DCs were incubated with increasing concentrations of the astragalus extract (A) and elderberry extract (B) and left unstimulated or stimulated with *E.coli* Nissle (10 µg/mL) or *L.acidophilus* NCFM (10 µg/mL). After 20 hr the cells were harvested and stained for viability with 7AAD for 15 min., washed and analysed by flow cytometry. (*: p<0.05; **: p<0.01; ***: p<0.001).

In contrast, astragalus root extract inhibited IL-1β production when added to *E. coli*-stimulated dendritic cells (Figure 2 C, p<0.05 with the dose 500 µg/mL astragalus root extract versus *E.coli* alone), whereas elderberry fruit extract did not affect such. IL-6 levels increased significantly, up to 2-fold, when astragalus root and elderberry fruit extract at the highest concentration (500 µg/mL) were incubated with *L. acidophilus*-stimulated dendritic cells (p<0.05). In *E. coli* stimulated dendritic cells, incubation with astragalus root or elderberry fruit extracts at the lowest concentration also increased by 2-fold IL-6 production (p<0.05). Increasing the concentration of the extract did not have a significant effect, suggesting active compounds with counteracting effects may have been present. The addition of the extract slightly affected the viability of the dendritic cells (figure 3). Both immature and bacteria stimulated cells had a viability of 86.5+/−2%. Addition of the extracts only slightly affected the viability of the *E.coli* and *L.acidophilus* stimulated cells (2.3–5.4% decrease in the highest concentration, while added in lower concentrations, a slight decrease in viability was only seen for elderberry and *L.acidophilus* stimulated cells (2.5%)). Interestingly, the extracts increased the viability of the unstimulated cells, even when added at the lowest concentration. Taken together, these results demonstrate that Astragalus root and Elderberry extract both modulated the cytokine responses to *E.coli* and *L.acidophilus* differently and that this is not due to toxic effects of the extracts.

We have demonstrated that much of the IL-12 that is induced by *L. acidophilus*, but not *E. coli*, is attributed to strong induction of IFN-β, which, by binding the IFN-α receptor (IFNAR), upregulates IL-12 and other genes that mediate viral defense and stimulation of Th1 responses. Thus, we hypothesized that the IL-12-enhancing effects of astragalus root and elderberry fruit extracts are the result of enhanced *Ifnβ* transcription.

**Figure 4:**
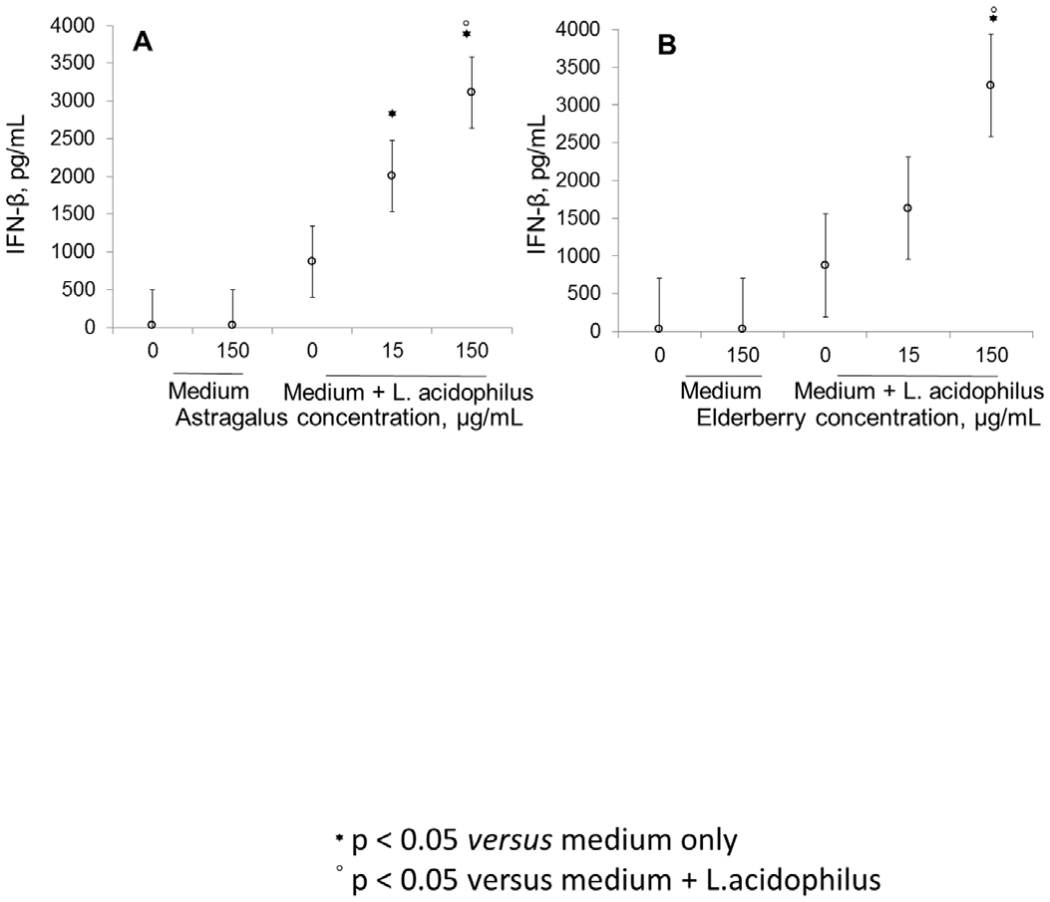
Astragalus and elderberry extracts potentiate the IFN-β-inducing capacity of *L. acidophilus* in dendritic cells. Extracts of astragalus root (A) and elderberry fruit (B) were added to murine bone marrow-derived dendritic cells at the concentrations 15 and 150 µg/mL prior to the addition of *L. acidophilus* (10 µg/mL) and incubated at 2, 4, 10, and 20 hr. Supernatants from the cells that were incubated for 20 hr were harvested, and IFN-β was measured by ELISA. Error bars are representative of the LSD intervals. The difference between 2 groups was considered significant when the LSD intervals did not overlap (p-value<0.05).

**Figure 5:**
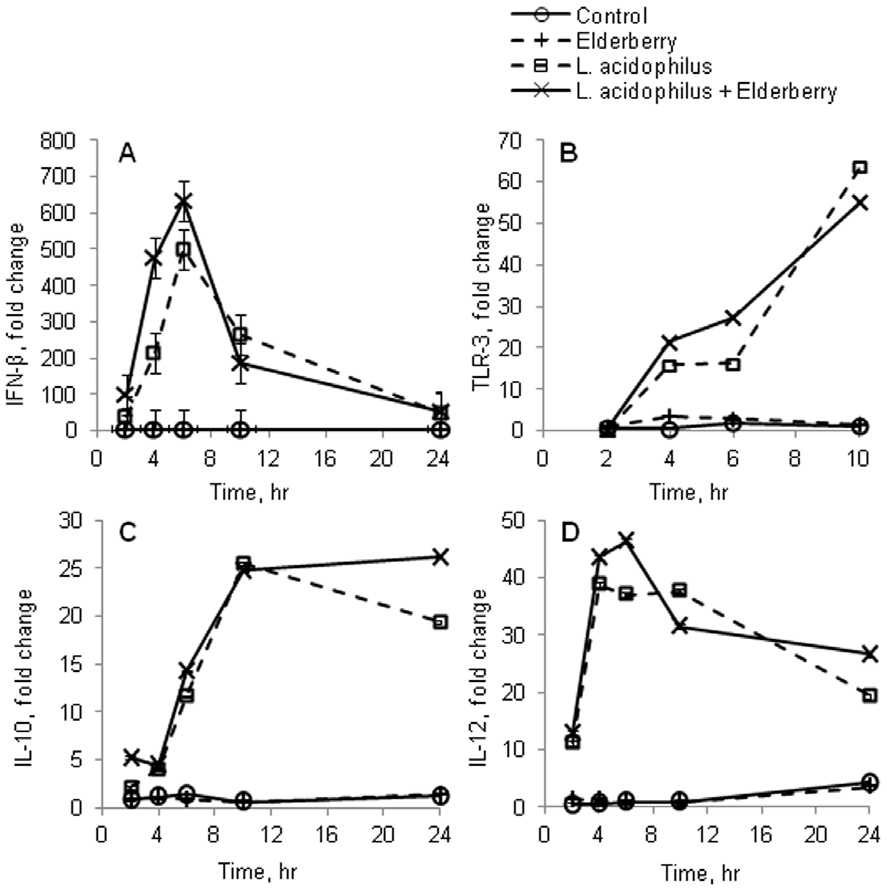
Elderberry extract potentiates *Ifn-β, Trl3, Il10* and *Il12* gene expression by *L. acidophilus* in dendritic cells. Extract of elderberry fruit was added to murine bone marrow-derived dendritic cells at the concentration 500 µg/mL prior to the addition of *L. acidophilus* (10 µg/mL) and incubated at 2, 4, 10, and 20 hr. RNA was harvested at various time points and analyzed for *Ifnβ*, (A), *Tlr3* (B), *Il12* (C) and *Il10* (D) expression by RT-PCR relative to β-actin. Cells only stimulated with *L.acidophilus* or elderberry extract were used as controls. Error bars are representative of the LSD intervals. The difference between 2 groups was considered significant when the LSD intervals did not overlap (p-value<0.05).

Astragalus root (Figure 4A) and elderberry fruit (Figure 4B) extract dose-dependently enhanced IFN-β levels, as measured in cell culture supernatants after 20 hr in response to *L. acidophilus* (p<0.05). *Ifnβ* transcription was unaffected by elderberry fruit extract alone but increased significantly when the extract was added to *L. acidophilus*-stimulated cells at 4 and 6 hr compared with *L. acidophilus* alone (p<0.05, Figure 5A). *Tlr3* expression rose at 4 and 6 hr in cells that were stimulated with elderberry fruit extract and *L. acidophilus* versus *L. acidophilus* alone; at 10 hr after stimulation, the expression in the 2 samples was comparable (Figure 5B).

Similarly, *Il10* (Figure 5C) and *Il12* (Figure 5D) expression increased significantly with the elderberry and *L. acidophilus* combination versus *L. acidophilus* alone, peaking at 6 and 24 hr, respectively. In summary, the increase in IFN-β demonstrates that astragalus root and elderberry fruit extracts contain components that upregulate *Ifnβ*, potentially enhancing the production of viral defense genes that are induced by the IFN-β/IFNAR pathway in response to *L. acidophilus*.

Immature dendritic cells constantly survey the environment by endocytosing external fluid. We examined the effects of elderberry fruit and astragalus root extract on the endocytotic activity of dendritic cells by measuring the uptake of FITC-conjugated dextran, which are inert nanoparticles that do not activate pathogen recognition receptors on dendritic cells, and *L. acidophilus*, the uptake of which is facilitated by ligation of bacterial components to various receptors on dendritic cells.

**Figure 6:**
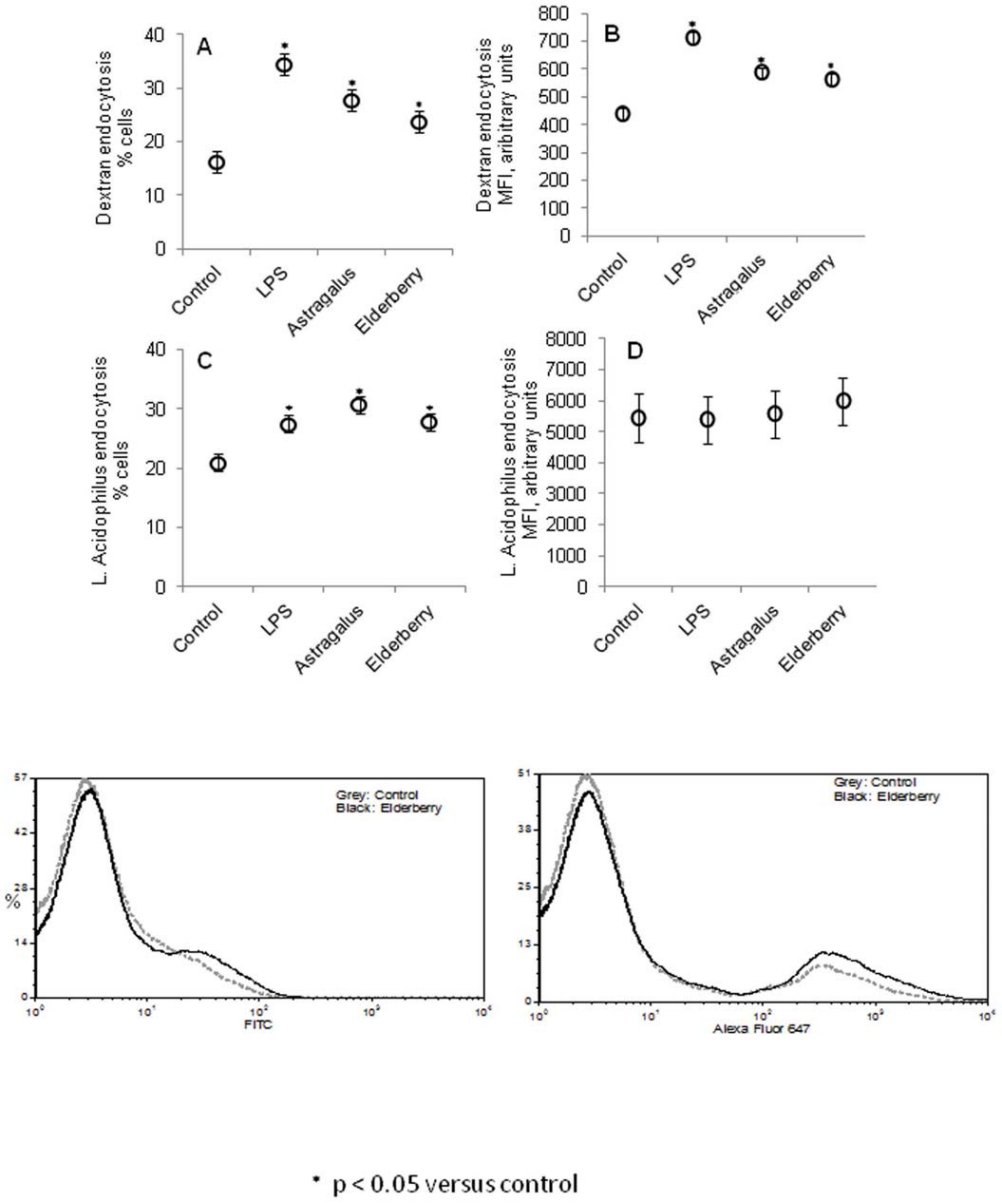
Elderberry and astragalus increase dextran and *L. acidophilus* uptake in immature dendritic cells. Immature murine bone marrow-derived dendritic cells were incubated with elderberry fruit extract, astragalus root extract, or LPS for 30 min prior to the addition of FITC-labeled 10 kD-dextran molecules (A, B) or Alexa Fluor 647-labeled *L. acidophilus* (C, D). Results are expressed as % of fluorescent cells (A, C) and as mean fluorescence intensity (MFI) (B, D). Error bars are representative of the LSD intervals. The difference between 2 groups was considered significant when the LSD intervals did not overlap (p-value<0.05). Lower panel show examples of histograms of which A–B were made; Fitc-dextran (left) or Alexa Fluor 647-conjugated L.acidophilus (right) incubated cells with or without prior incubation with elderberry fruit extract.

Elderberry fruit and astragalus root extract significantly enhanced endocytotic activity in unstimulated (immature) cells, as evidenced by uptake of FITC-conjugated dextran, versus control (p<0.05) (Figure 6A, 6B). This increase, however, was less robust than the effect of LPS. The addition of fluorescence-labeled *L. acidophilus* to cells that were incubated with elderberry fruit or astragalus root extract also increased the percentage of fluorescent cells (p<0.05) but not their mean fluorescence intensity (Figure 6C, 6D).

## Discussion

Certain foods and food components are traditionally used to maintain or improve immune activity, but their mechanisms of action remain to be determined. In this study, the modulatory effects of 2 botanical extracts on dendritic cells that were stimulated with *E. coli* or *L. acidophilus* were examined. Astragalus root and elderberry fruit extracts modulated immune cell responses, albeit disparately, depending on the bacteria. Both extracts enhanced *L. acidophilus*-induced production of IL-12 and mitigated IL-12 upregulation by *E. coli*. These results reflect the differential responses of these extracts to various microbes and demonstrate the Th1-skewing effects of astragalus root and elderberry fruit extracts. To our knowledge, this is the first time the effect of plant extracts on gram-positive and gram-negative bacteria stimulated dendritic cells has been compared and stresses the importance of the choice of stimuli for the outcome of such investigations.

Astragalus root and elderberry fruit extracts *per se* slightly upregulated the proinflammatory cytokines IL-6 and TNF-α in dendritic cells and elderberry fruit extract also induced IL-1β production. Generally, however, these responses are substantially lower than those to bacterial stimulation. Moreover, no IL-10 or IL-12 production was induced. As IL-10 and IL-12 are both induced in dendritic cells by the presence of even minor LPS contaminations [30], we can exclude significant LPS contaminations in the two extracts.

As demonstrated, the gram-negative bacterium *E. coli* induces IL-10 production and upregulates surface markers [31], whereas the gram-positive *L. acidophilus* induces IFN-β production, in turn upregulating genes that activate adaptive immunity and viral defense [3], [32]. Hence, disparate signaling pathways appear to be induced by the 2 bacteria, which might explain the differential effects of the extracts on the responses to the microbes. IFN-β has anti-inflammatory activity; type 1 IFN signaling through IFNAR and STAT1 inhibits IL-1β through upregulation of IL-10 production and repression of inflammasome activity [33]. In addition, IFN-β induces the production of IL-1β receptor antagonist *in vivo*, which inhibits IL-1β production [5]. The effects of the extracts on the cellular responses cannot be caused by a cytotoxic effect on the cells as the extracts only in the highest concentration showed a slight effect on the viability.

The viability of the cells was actually slightly increased in cells only treated with extracts and no bacteria, and cells stimulated with bacteria showed less viability, indicating that the presence of extracts or bacteria only have a minimal cytotoxic effect on the dendritic cells.

The response to *L. acidophilus*, but not *E. coli*, is associated with robust IFN-β synthesis [3]; thus, we expected to observe a reduction in IL-1β after adding IFN-β-enhancing extracts to *L. acidophilus*-stimulated cells. However, incubation of *L. acidophilus* in dendritic cells with the botanical extracts increased IFN-β, IL-10, and IL-1β production, indicating that IL-1β is induced by a different pathway on *L. acidophilus* stimulation that is not negatively affected by IFN-β or IL-10. Nevertheless, IFN-β production might still affect that of IL-1β by other cells.

The modulation of cytokine production by astragalus root and elderberry fruit extracts might be attributed to the activation of different signaling pathways in dendritic cells, the mechanisms of which are partially understood. Further, this immunomodulation might be caused by a specific component of the botanical extract or by synergistic effects of several components.

Although we did not address the identification of the active compounds in the present study it is tempting to speculate which compounds that might be involved. Polysaccharide fractions of astragalus root extracts contain bioactive components that influence the immune response [14]–[17]. Several nonstarch plant polysaccharides modulate dendritic cells. β-glucans and galactomannan guar gum synergistically increase IL-10 and TNF-α production and suppress IL12p70 on lipopolysaccharide (LPS) stimulation [30]. Thus, in our set-up, the combination of astragalus polysaccharides and E.coli, which containd LPS, might cause the IL-12 reducing effects. As regards the stimulating effect of the combination of astragalus extract and *L.acidophilus*, it was recently shown that the humoral immune response was enhanced upon administration of a combination of astragalus polysaccharides and lactobacillus [34]. Likewise, several immune modulating components in elderberry fruit extracts have been identified, including anthocyanins [21] and lectins [23], [25].

Identification of the active compound(s) of the two extracts, in particular of the IFN-β enhancing compounds, is however, not the scope of this study, which aimed to assess the effect of plant extracts on dendritic cells stimulated with intact bacteria. To this end, we have previously shown that in particular whole gram-positive bacteria induce distinct responses in dendritic cells as compared to bacterial ligands such as peptidoglycan and lipoteichoic acid [28]. Accordingly, our study is not comparable with previous studies using peptidoglycan or other bacterial cell wall constituents as stimulating ligands.

Overall, the 2 extracts have similar effects, despite their distinct content of putative bioactive compounds. In an initial screen, many other botanical extracts did not have immune-enhancing effects (unpublished data). Nevertheless, we cannot exclude the possibility that a specific component present in both extracts exerts the IFN-β enhancing effect, a hypothesis that remains to be examined.

Both extracts increased the uptake of the relatively small (10 kD) and inert dextran particles, as well as whole bacteria that express ligands for TLRs and other pathogen recognition receptors. But, whereas LPS, which enhances endocytotic activity in dendritic cells [35], had a stronger effect on the endocytosis of dextran, the 2 extracts were as potent as LPS in increasing the uptake of *L. acidophilus*. This enhancement manifested as an increase in the number of cells that took up fluorescently labeled bacteria, whereas that of dextran was observed as increases in the number of cells and the amount of endocytosed dextran. This disparity might be explained by the underlying endocytotic mechanisms of such diverse entities as dextran and *L. acidophilus*. In accordance with our previous findings of the importance of the endocytotic pathway for the cytokine response [3], these data could indicate that the extracts affect the cytokine signaling by affecting the uptake of *L.acidophilus*.

We used extract concentrations in the range of 15–500 µg/mL, but as these commercial extracts were stabilized with either maltodextrin (elderberry) or gum arabicum (astragalus root), the real extracts concentration used in the cell experiemens were actually 7–250 µg/mL(astragalus root) and 5–170 µg/ml (elderberry fruit), respectively. The two carrier materials did not *per se* affect the cell responses (data not shown). In general, we found that extract concentrations down to around 20–25 µg/mL excerted significant effects on the cytokine production. Whether these concentrations are too high to be realistic *in vivo* cannot be concluded as long as the concentration of the active compound(s) is not known. Although we acknowledge that the bioavailability of the active compounds are of major importance in order to be able to understand the impact of our presented data, the identity of the compounds as well as the amounts present in the extracts have to be established before we can relate our results to *in vivo* conditions.

In conclusion, astragalus root extract and elderberry fruit extract enhance the production of Th1-skewing cytokines, IL-12 and IFN-β, in murine bone marrow-derived dendritic cells that have been stimulated by *L. acidophilus* but not *E. coli*. This is the first study to demonstrate distinct immunomodulatory effects of plant extract in combination with stimulating bacteria and also the first report of the enhancing activity of plant extracts on the *L.acidophilus* induced IL-12 and IFN-β production. These extracts may find clinical use in boosting immunity and preventing viral infections, but the active compounds remain to be identified.
